# Performance Tests of HX340 Microalloyed Steel Sheets Joined Using Clinch-Rivet Technology

**DOI:** 10.3390/ma17030596

**Published:** 2024-01-26

**Authors:** Łukasz Boda, Jacek Mucha, Waldemar Witkowski

**Affiliations:** 1 Doctoral School of the Rzeszow University at Technology, Al. Powstancow Warszawy 12, 35-959 Rzeszow, Poland; d413@stud.prz.edu.pl; 2 Faculty of Mechanical Engineering and Aeronautics, Rzeszow University of Technology, Al. Powstancow Warszawy 8, 35-959 Rzeszow, Poland; wwitkowski@prz.edu.pl

**Keywords:** clinch-rivet, HX340 steel joining, joint strength, interlocking joints, sheet deformation

## Abstract

This paper presents the possibility of forming a pressed joint between two 1.5 mm thick sheets made of HX340 steel. The joint was formed using an additional deformable steel rivet with a hardness of 400HV1. Different distances of rivet pressing below the initial surface of the upper sheet were chosen: 0, 0.15, 0.30, 0.45 and 0.60 (in mm). For the mentioned rivet-pressing distances, forming tests were performed using an “SKB” die with three die depths: 1.45, 1.60 and 1.75 (in mm). Observations and measurements of the characteristic geometric dimensions of the interlock were made. Furthermore, the strength of the clinch-riveted joints was analysed. Changing the depth of the die by 20%, i.e., from *h_d_* = 1.45 mm to *h_d_* = 1.75 mm for the same rivet press-in depth (*o_f_* = 0%), resulted in a 12% reduction in the forming-force value. The maximum load capacity of the joint in the shear test decreased by 3.5% for the mentioned joint variant. The greater the rivet press-in depth used in the joint (*o_f_*), the greater the value of the interlock size (*t_u_*). Increasing the value of rivet-pressing depth below the initial surface of the upper sheet from 0 mm to 0.6 mm resulted in an increase in the size of the interlock (*t_u_*) by 136% (for *h_d_* = 1.45 mm), by 128% (for *h_d_* = 1.60 mm) and by 85% (for *h_d_* = 1.75 mm).

## 1. Introduction

Until recently, techniques based on resistance welding, welding or laser welding were the most common methods of joining car body elements. Due to the fact that these processes generate harmful gaseous compounds and mechanically and thermally influence the material structure of the joined elements, other joining processes have been developed. For some time, the clinching process with an additional rivet and self-piercing riveting has been one of the methods more frequently used than welding [1,2]. The mentioned joining techniques make it possible to obtain a joint with relatively high stiffness and strength compared to clinch joints (“CL”) [3]. Clinching can be used to join galvanized sheets or sheets with other coatings [4]. Clinching technology can replace resistance welding in some cases [5]. Clinching technology has such great potential that it is constantly being developed [6]. Modifications to this technology are being introduced, such as additional local heating of the materials before joining [7] or welding the bottom of the embossment [8]. Another modification of clinching joining technology is joining sheets with a hole already made in the lower layer [9]. This method is suitable for joining composites with metals. One of the other methods of connecting pieces without the need to make holes is self-piercing riveting (“SPR”) [10,11]. In the “SPR” joining process, the upper sheet is pierced. Hole-less riveting can be used to join together various metal materials, including aluminium alloys, but also to join together various construction materials, e.g., various metal compositions [12,13] or titanium alloys [14,15].

When joining using the clinch-riveting method, the continuity of the material of the joined sheets is maintained, and there is no intentional puncture of the joined material layers. As a result of the use of an additional rivet to form the clinch joint, a relatively large increase in the maximum load capacity is achieved [16,17,18]. Another way to strengthen the clinch joint is to use additional adhesive in the process of assembling the sheet-metal elements [19].

The formation of clinch-rivet joints can be performed using full deformable steel rivets of various hardness values [20]. The authors of work [20] presented the results of research on the influence of different hardness values of rivets (350HV1, 400HV1, 420HV1) on the forming force, geometric parameters and load capacity of the joint for steel sheets.

In the case of selected materials, it is also possible to join them with an additional rivet (without damaging the joined sheets) using a die with a flat surface. In [18], a flat die was used to join aluminium alloy sheets. An analysis of the influence of the blank holder force on the geometric parameters and the load capacity of the joint was presented.

A full rivet can be used to repair a clinch joint with damage to the joined sheets in the embossment area [21,22]. A rivet with a hole makes it possible to reduce the energy consumption of the forming process while maintaining a high load capacity for the joint [23]. Paper [23] presents the influence of different diameters of the hole in the rivet on the formation and load capacity of the lap joint of DX51D steel sheets. The lowest forming force was observed for a through hole with a diameter of 2.5 mm. “CR” joints were formed using rivets made of materials with different hardness values.

A rivet made of aluminium alloy with a hole can also be used to strengthen a damaged clinch joint [24]. In [25], the authors presented research on joining using a solid rivet and a hole rivet in a two-stage forming process. It is also possible to completely simplify the shape of the die and use a special blank holder in the process of pressing the rivet into the joined materials [26]. Another method of joining using a full rivet was presented in works [27,28,29,30]. To join layers of different materials, a high-hardness rivet was used. The rivet punched a hole, and afterward, it was blocked with the joined material.

All modifications and innovations in pressure joining technologies are part of one of the trends in the automotive industry [31,32,33]. Clinching technology can be used to join three layers of sheets [34]. It should be mentioned that each method of joining thin-walled elements causes deformation of the material around the joining point. During joining, there is a local change in the structure of the material, and there is a specific deformation of the surface of the joined sheets [35].

This work presents research, results and analysis of the possibility of using a steel deformable rivet with a hardness of 400HV1 as an additional element for forming clinch joints between sheets made of microalloyed steel and F-type zinc coating (ZiNc). A full solid rivet was used to form the clinch-rivet joints. The rivet was pressed to different depths (offsets) relative to the upper surface of the sheets using an ‘SKB’ die. Forming with different offsets was carried out for three different ‘SKB’ die depths with movable and fixed segments. In addition, the energy consumption of the forming process was analysed for various die depths. The basic geometry of the cross-sections of the joints was measured, and the deformation of the sheets was measured in the case of the highest observed values of sheet deviation, i.e., for the case of matrix depth *h_d_* = 1.45 mm. The load capacity of the joints was also tested for a lap joint made of 1.5 mm thick HX340 steel sheets.

## 2. Materials and Methods

### 2.1. Materials

Microalloyed steel, produced at the U. S. Steel Košice, s.r.o. plant (Košice, Slovak Republic), which is increasingly used for structural elements of car bodies, was used in experimental tests. The thickness of the sheets was 1.5 ± 0.11 mm (according to the EN 10143/06 standard [36]), and the material nomenclature was HX340LAD+Z (EN 10346/09 standard [37]); the sheets had a ZiNc coating (100 g/m^2^) with an average thickness of 8 μm (according to the EN 10346/09 standard). The basic chemical composition and mechanical properties are presented in Table 1 and Table 2.

The deformable steel rivets, type A5x5-2Al, used in the “CR” joining technology had an average hardness of 400HV1. The characteristics of the rivets have been presented in [20,23]. Hardness measurement (average of 5 measurements) using the Vickers method was performed using a Matsuzawa microhardness tester, type Micro-Sa, Seiki Co., Ltd. (Nagaoka-shi, Japan), at a load of 10 N (in accordance with the ISO 6507-1:2018 standard [38]).

### 2.2. Mechanical Joining Process

**Figure 1:**
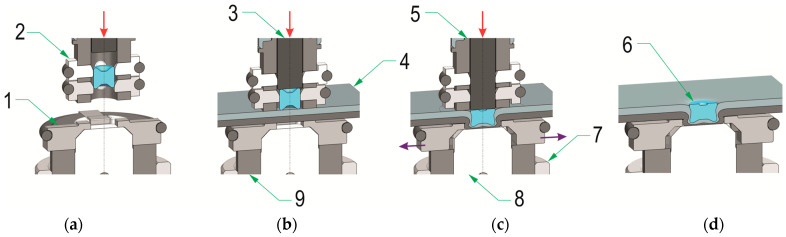
The main phases of the sheet joining process: (**a**) positioning the rivet in the clinch-riveting punch system before placing the sheets for joining—phase I, (**b**) pressing the rivet into the joined layers of materials—phase II, (**c**) final pressing of the rivet against the bottom of the die—phase III, (**d**) clinch-rivet joint after retraction of the clinch-riveting punch system; 1—SKB die with movable segments, 2—sliders in the punch system, 3—punch, 4—joined sheets, 5—bush of the punch system, 6—rivet after forming process, 7—bush of the die system, 8—pin of the die system, 9—bush die with movable segments, red arrow—direction of the punch movement, purple arrow—direction of the die sliding segments movement.

The joints were formed in the Pressed Joints Laboratory of the Machine Design Department at Rzeszow University of Technology (Rzeszow, Poland). The forming press consisted of a C-shaped frame and an EMPK electric drive system (Tox Pressotechnik, Wrocław, Poland) with a maximum pressing force of 100 kN. The press measuring system is characterized by a displacement measurement accuracy of 0.01 mm. The maximum force measurement error was not greater than 0.5% of the actual pressing force. The joint forming process consisted of feeding the rivet from the clinch-riveting punch system and then moving it to the punch bush (Figure 1). The joints were formed using a special rivet punch system with an automatic mechanism to insert the rivet into the space in front of the pressing punch. The rivet was positioned in front of the pressing punch on the appropriate sliders placed in the holes in the bush. Then, the rivet was gradually pressed into the joined sheets. After the bottom sheet came into contact with the bottom of the die, due to the pressure of the punch on the rivet, the rivet material flowed more intensively in the radial direction. The rivet and sheet material filled the space of the movable segments of the die. After the face of the rivet-pressing punch reached a predetermined position, the punch system was withdrawn and the joint was formed. The position of the rivet was controlled with an accuracy of 0.01 mm.

**Figure 2:**
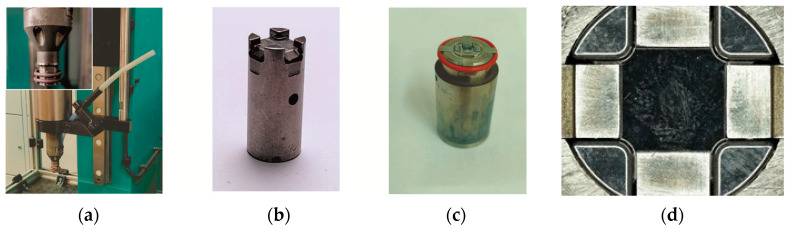
The set of tools used for the experimental formation of “CR” joints: (**a**) the clinch-riveting punch system, (**b**) example of the interchangeable pin, (**c**) bush die with pin and movable segments, (**d**) top view of die with flat bottom.

**Figure 3:**
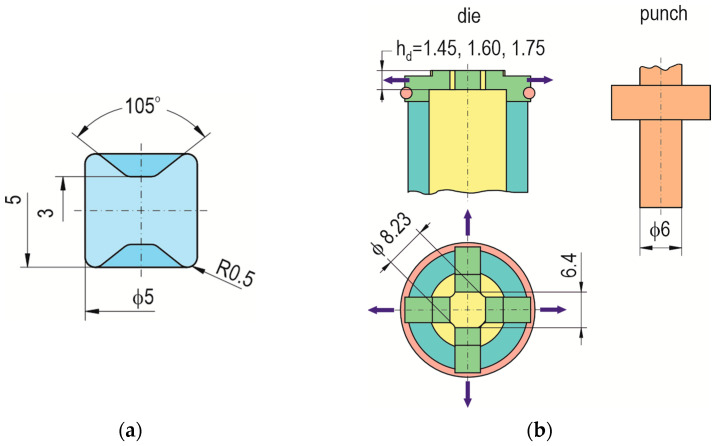
The basic geometry and dimension of (**a**) the rivets, (**b**) the die and rivet-pressing punch (in mm), purple arrow—direction of the die sliding segments movement.

**Figure 4:**
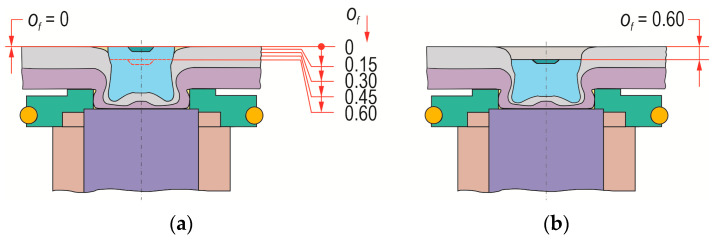
The relative position of the upper surface of the rivet in the joint: (**a**) rivet’s final position for offset *o_f_* = 0, (**b**) rivet’s final position for offset *o_f_* = 0.6 (*o_f_* in mm).

For research on the formation of “CR” joints between two HX340 steel sheets, a die with 3 depths—*h_d_* = 1.45, 1.60 and 1.75 (in mm)—and a flat bottom was used (Figure 2). Forming was carried out using a clinch-rivet punch system (Figure 2a) and a developed replaceable die system (Figure 2b,c) allowing easy replacement in the bush holder (Figure 2c) and mounting on the press stand. The characteristic shape and dimensions of the rivet, punch, and dies are shown in Figure 3. First, basic joints were made for the upper surface of the rivet to obtain a position corresponding to the initial position of the upper surface of the sheets. Then, for each depth of the die, clinch-rivet joints were formed for additional 4 offset values, namely, *o_f_* = 0.15, 0.30, 0.45 and 0.60 (in mm), as shown in Figure 4a. An example of a joint with a rivet offset *o_f_ =* 0.6 mm is presented in Figure 4b. All tested variants of the “CR” joint are listed in Table 3.

**Figure 5:**
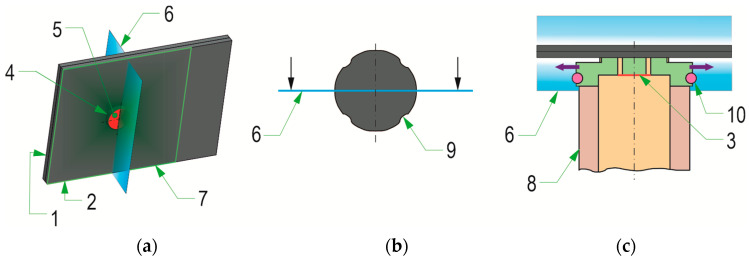
The characteristics of (**a**) the base external surface of the lower sheet, (**b**) the transverse plane of the joint cross-section, (**c**) the initial position of the sheets in relation to the bottom of the die; 1—upper sheet metal, 2—lower sheet metal, 3—surface of the bottom of the die (reference surface for the position of the sheets), 4—embossment, 5—outer lower surface of the embossment, 6—cross-sectional plane of the joint, 7—boundaries of the scanned area of the upper and lower surfaces of the joint, 8—die with movable segments, 9—places of interaction of fixed segments of the die, 10—spring element, black arrows—direction of the joint cross-section, purple arrow—direction of the die sliding segments movement.

**Figure 6:**
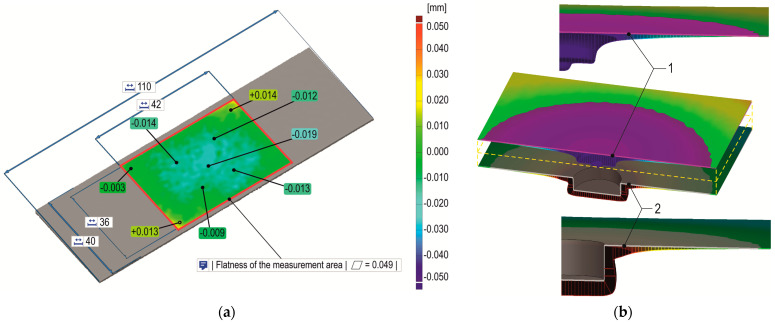
The characteristics of (**a**) the flatness of the base surface of the sheets before joining (in mm), (**b**) a comparison of the 3D scan model with the base model of the location of the characteristic joint surfaces (1—upper base surface, 2—lower base surface).

Joint cross-sections were prepared. It was observed that the greatest deviation of the sheets occurred for the case of die depth *h_d_* = 1.45 mm. The cross-section of each joint was made in the joint axis so that the cross-sectional plane intersected the material that had flowed between the fixed segments of the die (Figure 5b). Therefore, joint samples were again taken according to the diagram in Figure 5a, and it was decided to scan the external surfaces of the sheets. The surface of the bottom of the die, surface “3”, (Figure 5c) corresponded to surface “5” of the embossment of the nominal CAD model (Figure 5a). On the basis of the location of the external surfaces of the sheets in the nominal CAD model, the base reference planes were determined to analyse the deviation of the external surfaces of the joint samples. The initial surface of the sheets was measured; on this basis, a nominal model of the sheets was established, and the base (initial) system of the reference surface was determined (Figure 6a). After the base surfaces of the sheets and the nominal locations of the rivet-face and die-bottom surfaces were applied, a nominal comparative CAD model was obtained. After measurement on a 3D scanner, a model of the deformed sample was obtained. Then, both models were compared with a common coordinate system (Figure 6b).

The surface deviations of the sheet metal were measured using an ATOS Capsule 200 MV200 scanner (Carl Zeiss Sp. z o.o., Warsaw, Poland). The measurement system was determined by parameters obtained through the acceptance test according to guideline VDI/VDE 2345 Part 3 [39], in line with the GOM Acceptance Test. The measurement system was characterized by the maximum measurement error: sphere spacing error 0.008 mm and length measurement error 0.009 mm. After the scan, points along the path were set, and deviations were visualized in the form of an area distribution.

**Figure 7:**
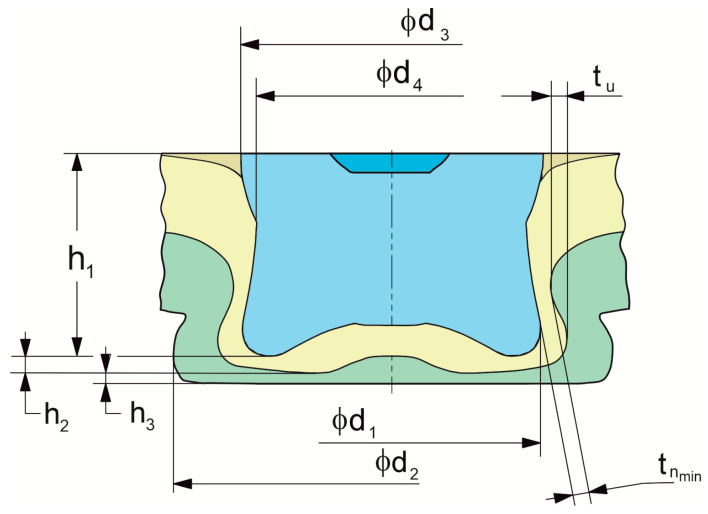
The basic interlock parameters of the “CR” joint.

For all variants of the joints (Table 3), observations and measurements of characteristic parameters of the joints’ interlocks were made (Figure 7). Macro photos and joint geometry measurements were taken using the VHX7000 optical microscope (KEYENCE INTERNATIONAL, Mechelen, Belgium). The microscope was equipped with a VH-Z20R/Z20T zoom lens (KEYENCE INTERNATIONAL, Mechelen, Belgium). The resolution of a single image was 2048 × 1536 [px], and the distance between the pixels of the recorded image, in both directions of the XY plane, was 0.0015 mm.

**Figure 8:**
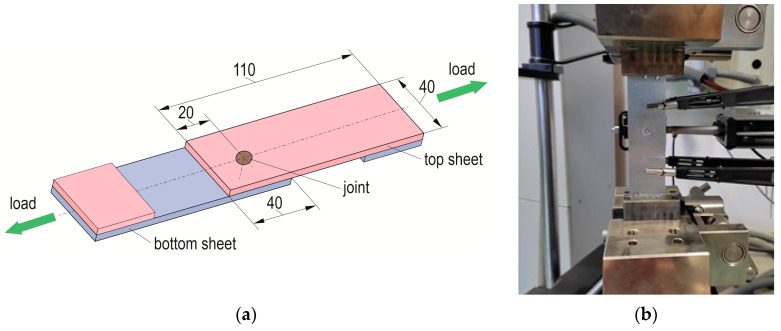
The “CR” lap-joint samples: (**a**) dimensions (in mm), (**b**) mounted on the tensile test machine with an extensometer system.

To analyse the load capacity of the clinch-rivet joints, samples of lap joints, made of sheets with dimensions of 40 × 110 mm, were used. The geometry of a lap joint is shown in Figure 8a. The dimensions were determined based on the guidelines given in the ISO 12996: 2013 standard [40]. For each arrangement of lap joints (according to Table 3), seven samples were made. Strength tests of the lap joints were carried out on a tensile test machine with a measuring system of 100 kN (Figure 8b).

## 3. Results and Discussion

### 3.1. Joint Forming Process and Interlock Parameters

**Figure 9:**
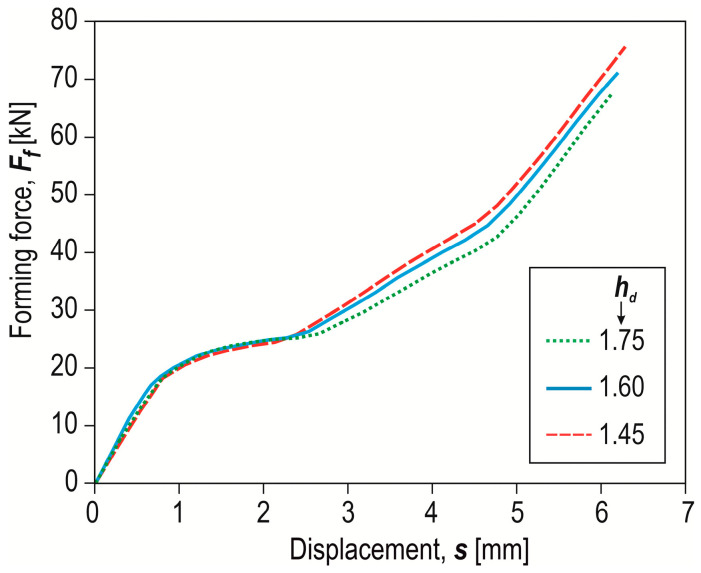
Examples of forming force–displacement diagrams for “CR” joining with a die with three values of depth (*h_d_* in mm).

**Figure 10:**
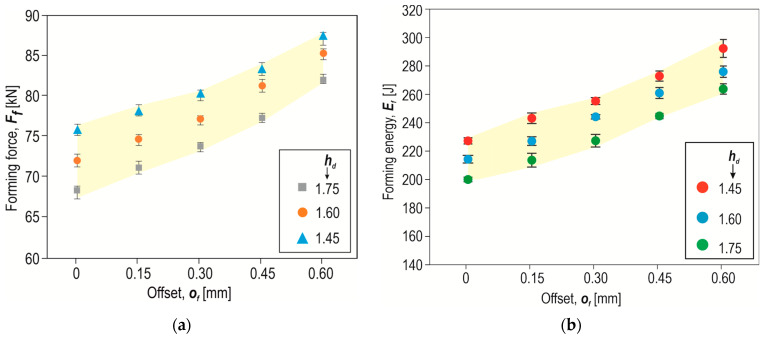
The influence of the offset value *o_f_* on (**a**) the forming force *F_f_*, (**b**) forming energy consumption *E_f_* (*h_d_* in mm).

The formation of a “CR” joint using a die with a depth of *h_d_* = 1.45 mm and an offset *o_f_* = 0 mm resulted in the highest forming force being required (Figure 9). For the remaining two die depths, a lower forming force was obtained, with the forming force for *h_d_* = 1.75 mm being the lowest. For each value of the depth of the die, increasing the offset value resulted in an increase in the forming force (Figure 10a). For the case of offset 0 mm, the impact of the depth of the die was relatively large. Increasing the depth of the die from 1.45 mm to 1.75 mm (by nearly 20%) resulted in a 12% reduction in the forming force. The higher the offset value below the upper surface of the sheet metal, the smaller the observed differences in the forming force were. In addition to changing the depth of the die, the energy consumption of the “CR” joining process can be reduced by using a modified rivet [23].

The smallest influence of the die depth on the forming force was observed for the largest offset, *o_f_* = 0.6 mm. A higher value of the offset *o_f_* increases the force loading of the tools. The loads of the tools influence their deformation and wear [41,42]. The highest energy consumption in the forming process was observed for the case where *o_f_* = 0.6 mm (Figure 10b). However, the greatest reduction in forming energy consumption was obtained for the case where *o_f_* = 0.15 mm and where the depth of the die was increased from *h_d_* = 1.45 mm to 1.75 mm.

**Figure 11:**
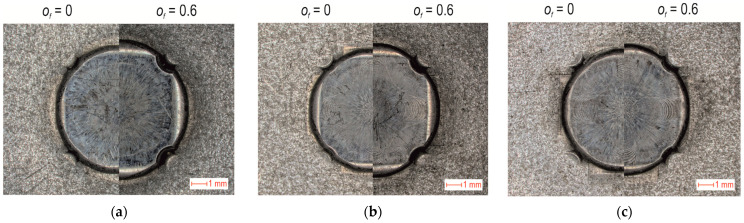
View of the embossment from the die side of the “CR” joint at the following die depths: (**a**) *h_d_* = 1.45, (**b**) *h_d_* = 1.60, (**c**) *h_d_* = 1.75 (in mm).

**Figure 12:**
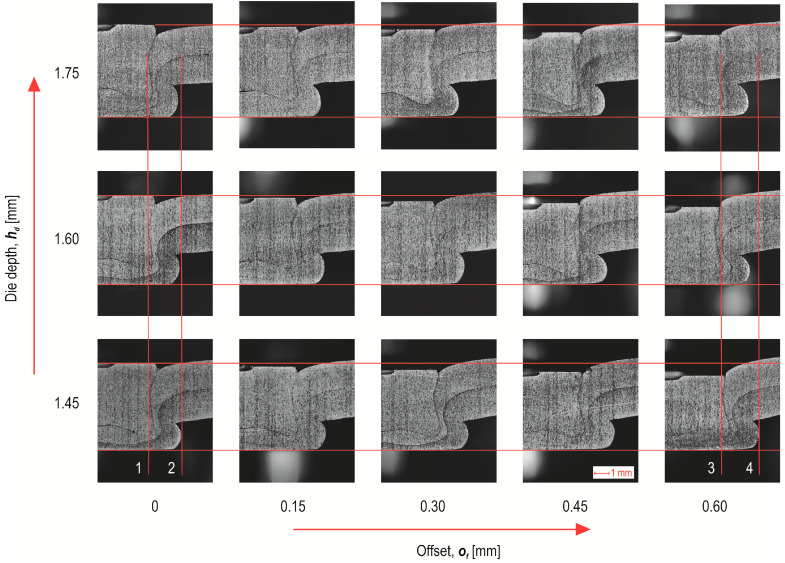
The “CR” joint cross-sections on macro scale (in mm).

In Figure 11, the embossment of the material pressed into the die groove is presented. In all the cases of *h_d_* values, the largest outer diameter of the embossment was obtained for the largest offset (Figure 11). The largest difference in maximum diameter of the embossment between offset 0 mm and 0.6 mm was observed for the case of forming the joint with a die with *h_d_* = 1.45 mm. The smaller the die depth, the more the fixed die segments influenced the material so that it flowed more intensively, moving the movable segments. The larger the diameter *d*_2_, the more intensely the sheet material was pressed between the fixed segments. For the offset *o_f_* = 0 mm, the differences were insignificant. The more the offset was increased, the greater the differences. Hence, the pictures show changes in the structure and shape of the joint in cross-sections (Figure 12). It is possible to reduce embossment height by joining with an additional rivet (without damaging the joined sheets) when using a flat die [18]. In [18] it was shown that the higher the pressing force of the sheets, the greater the geometric parameters and load capacity of the joint. A solid and nondeformable rivet (of high hardness) was used in their research.

**Figure 13:**
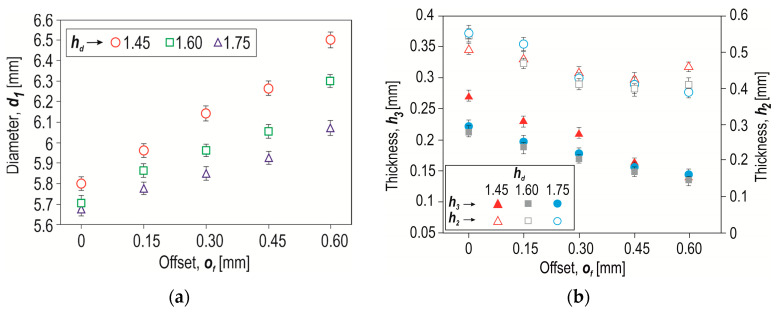
The influence of the depth of the die (*h_d_*) and the offset value (*o_f_*) on (**a**) the maximum diameter of the rivet—*d*_1_, (**b**) the minimum thickness of the sheet in the interlock—*h*_2_ and *h*_3_.

The higher the offset value, the greater the intensity of the sheet material flow and thus the greater the maximum diameter *d*_1_ of the rivet in the joint (Figure 13a). The greatest impact of the depth of the die on the diameter *d*_1_ was obtained for the largest offset of 0.6 mm. The highest minimum thickness of the lower sheet in the interlock (*h*_3_) was for the case of forming a joint using a die with a depth of *h_d_* = 1.45 mm—Figure 13b. Increasing the depth of the die from 1.45 mm to 1.75 mm caused the dependence of the minimum thickness of the lower sheet to change for an offset greater than 0.45 mm. In the case of *h*_3_ thickness, the larger the offset used, the lower the value of this parameter was obtained. The decrease in this value (*h*_3_) was not the same as for the thickness parameter *h*_2_. In the case of *h*_2_, the decrease in value as the offset increased was not as in the case of *h*_3_. Despite the decrease in the value of *h*_3_ as the offset increased, an increase in the value of the parameter *h*_2_ was observed at an offset greater than 0.45 mm. It should be mentioned that in none of the cases analysed did a puncture of the two joined layers of sheet metal occur.

**Figure 14:**
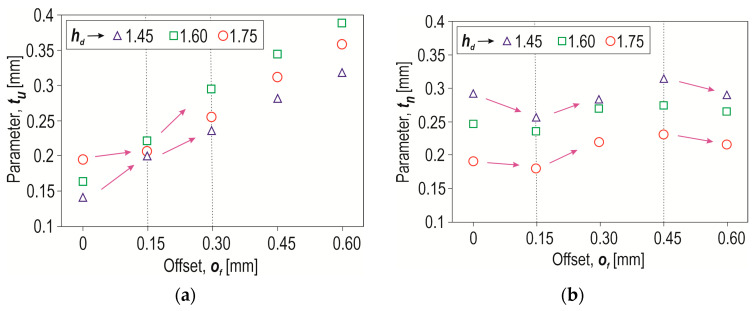
The influence of the depth of the die (*h_d_*) and the offset value (*o_f_*) on the interlock parameters: (**a**) *t_u_*, (**b**) *t_n_*.

It was also noticed that the offset value significantly influenced the values of the interlock parameters *t_u_* and *t_n_*. When joining high-strength steels, it is not always possible to form an interlock, even with the use of the SPR or clinching method [43,44]. Therefore, the authors of this publication investigated the influence of the change in the depth of insertion of the rivet (*o_f_* = 0–0.6 mm) and the depth of the die (*h_d_* = 1.45–1.75 mm) on the formation of the interlock. For all three depths of the die (*h_d_* = 1.45, 1.60 and 1.75 mm), it was observed that at an offset of 0.15 mm, there were characteristic values of the interlock parameter *t_u_* (Figure 14a). For an offset greater than 0.15 mm, there was a significant increase in the parameter *t_u_*. For each value of die depth, an increase in the interlock parameter *t_u_* was obtained from the offset value of 0.15. The greatest difference in the impact of the depth of the die on the interlock parameter *t_u_* occurred for an offset of 0.6 mm.

Increasing the value of the offset from 0 mm to 0.6 mm resulted in the greatest increase in the value of the interlock (*t_u_*). In the case of a die with a depth of *h_d_* = 1.45 mm, the increase was 136%; for *h_d_* = 1.60 mm, the increase was almost 128%; and for *h_d_* = 1.75 mm, the interlock value increased by 85%.

In the case of a minimal thickness of the interlock *t_n_*, the greatest impact of the parameter *h_d_* occurred for an offset of 0 mm (Figure 14b). In the range of offset values from 0.15 mm to 0.45 mm, both *t_u_* and *t_n_* increased (for all depths of the die). Increasing the offset value from 0.45 mm to 0.6 mm resulted in an increase in the parameter *t_u_* and a decrease in the parameter *t_n_*. This happened because the rivet bent the material of the lower sheet so that the size of the interlock increased and the sheet-metal thinning in the range of 0.15 mm to 0.45 mm decreased. Thus, the parameter *t_n_* increased. Only at offset *o_f_* = 0.6 mm did the rivet deformation cause thinning of the interlock, such that the value *t_n_* decreased (for all values of the depth of the die). The use of a rivet with greater hardness can increase the interlock parameters and increase the load capacity of the clinch-rivet joint [20].

### 3.2. Rivet and Sheet Deviations

**Figure 15:**
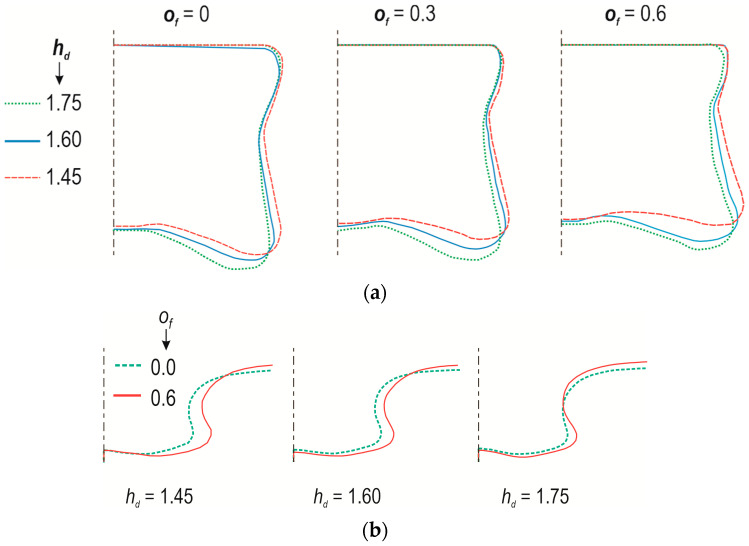
A comparison of the interlock shape for different values of the depth of the die (*h_d_*) and the offset value (*o_f_*): (**a**) for rivet, (**b**) for line between lower and upper sheets.

**Figure 16:**
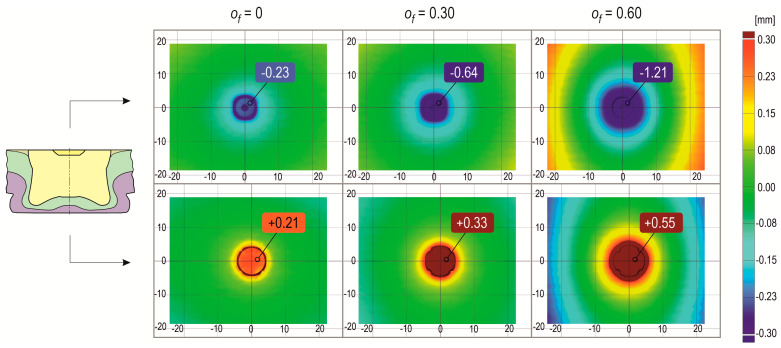
The influence of the offset value (*o_f_*) on the sheet deformation in the join’s axis area (*h_d_* = 1.45 mm).

An additional deformable element in the form of a solid rivet indirectly forms an interlock between the joined sheets. To characterize the influence of the offset value and the depth of the die on the deformation of the rivet, its interlock outlines were compared (Figure 15a). Proportionally to the deformation of the rivet, a common internal line of two sheets was formed (Figure 15b). To properly form a sheet interlock, the rivet must fill the space in the die cavity. The highest radial flow of the rivet material was observed in the case of die *h_d_* = 1.45 mm and the maximum offset (*o_f_* = 0.6 mm). In this case, the rivet was most compressed, causing its material to flow in the radial direction (Figure 15a). It was noticed that for this case (a joint formed with a die depth *h_d_* = 1.45 mm), the greatest deviation of the sheet surfaces around the joint occurred (Figure 12). Therefore, it was decided to measure and analyse the deformation of the upper and lower sheets. Figure 16 shows the results representing the deviations of the upper and lower surfaces of the joined sheets from the base surfaces of the sheets (before joining).

**Figure 17:**
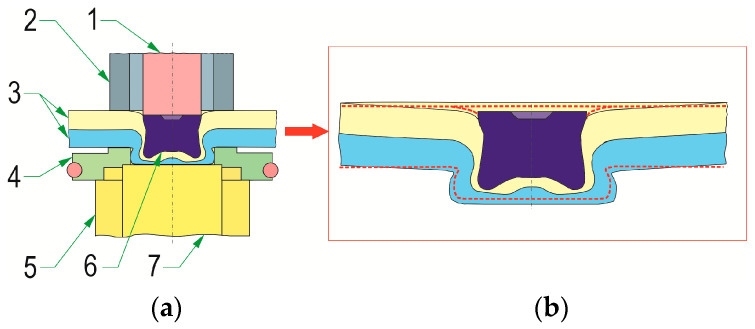
The schematics of (**a**) the blank holder acting during the formation process, (**b**) the effect of the sheet material relaxation after the tools were withdrawn (dashed line—outline of the joint before tool retraction); 1—punch, 2—blank holder, 3—sheets, 4—“SKB” die with movable segments, 5—bush die with movable segments, 6—rivet, 7—pin of the die system.

**Figure 18:**
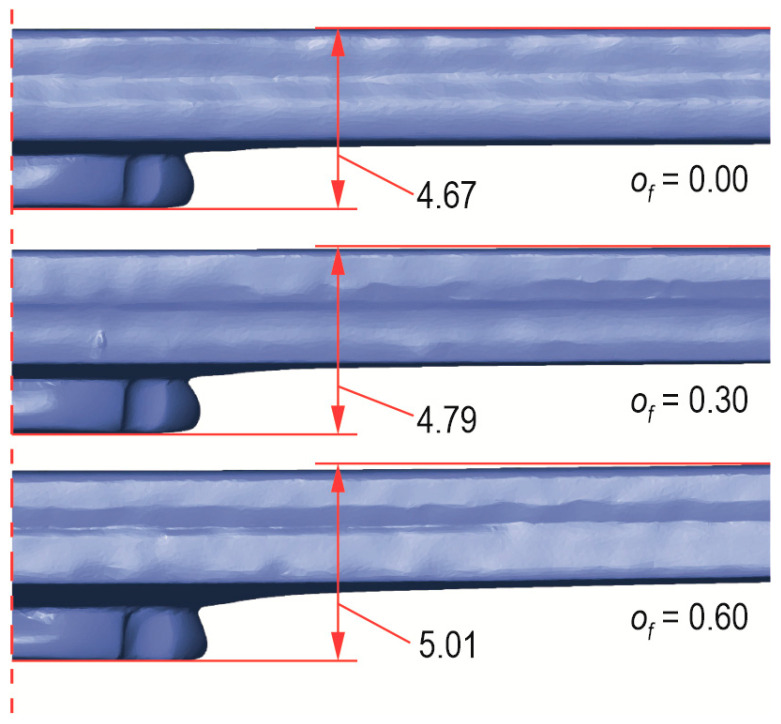
Examples of the influence of the offset value (*o_f_*) on the sheet deviations—*h_d_* = 1.45 mm.

The presented distributions of deviations on the sheets resulted from the fact that a rivet was pressed into the sheets. The sheets were pressed into the die cavity. The higher the offset values, the greater the deviations from the base plane of the sheets. In the case of deformation of the upper and lower sheets, the deviations in the position of the external surfaces varied from positive to negative values. During the forming process, the offset value was controlled with an accuracy of 0.01 mm. After forming the joint (Figure 17a) and withdrawing the tools, the existing stresses in the deformed sample elements caused them to relax (Figure 17b). The total deviations in the finished joint around the joint were a superposition of the deviations resulting from the phenomenon of material relaxation and the deformation resulting from the pressing of the rivet into the sheets. The existing stresses caused relaxation of the surfaces of the joined elements. As a result, the values of deviations from the nominal model changed—for the sheets from their initial position, as well as for the front surface of the rivet and the outer surface of the embossment from the position set by the forming tools. In the case of the connection for *o_f_* = 0 mm, the rivet face was below the reference plane by 0.23 mm. For the join with *o_f_* = 0.3 mm, the rivet face was below the reference plane by 0.64 mm. Finally, in the case of the joint for *o_f_* = 0.6 mm, the rivet face was below the reference plane by 1.21 mm. The difference between the offset value in the forming process and the measured position of the rivet face (relative to the base plane) is the springback value of the joined sheets. Examples of the total deviation of the planes of the joined sheets from their base position are shown on the 3D scan model of the “CR” joint (Figure 18). In each of the cases presented in Figure 18, the nominal value of the distance between the upper and lower surfaces of the joint should be 4.45 mm. The higher the offset value, the greater the difference between the upper and lower surface distances. Deformations of the joined elements around the joint are extremely important when joining thin-walled elements together. It is necessary to maintain the assumed nominal parameters of the shape and dimensions of the part. The method of predicting the surface deviation of aluminium alloy elements joined using “SPR” is presented in [45]. During the tests, the influence of the rivet height on the joining process and the deformation of aluminium alloy sheets was analysed.

### 3.3. Strength and Failure Mechanism of the “CR” Joint

**Figure 19:**
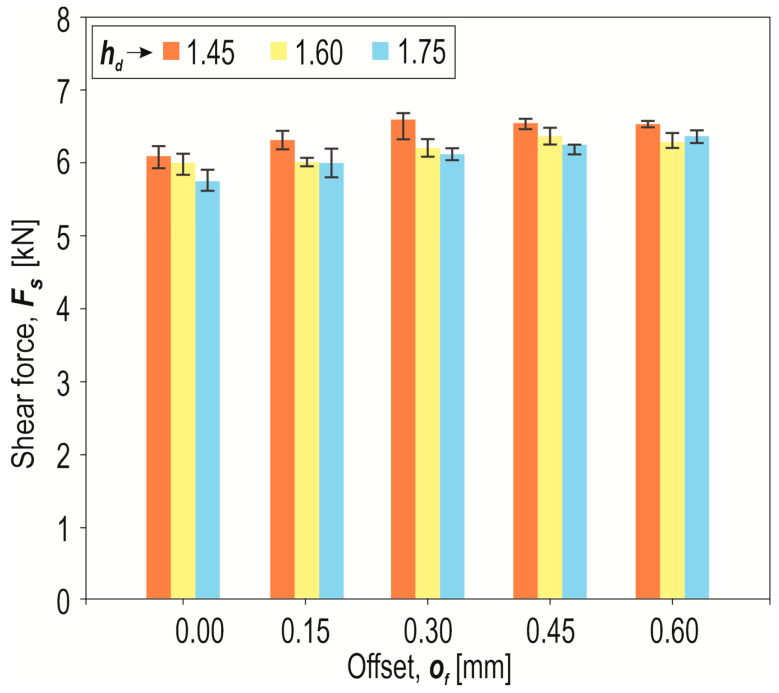
The influence of the depth of the die (*h_d_*) and the offset value (*o_f_*) on the maximum shear force during tensile shear tests of “CR” joints.

A certain regularity was observed for all joint variants—the smaller the depth of the die, the greater the maximum shear force of the lap joint (Figure 19). For each of the variants of the joint formed using a die of three different depths, an increase in shear force was obtained as the offset value increased. The use of a die (*o_f_* = 0 mm) with a greater depth than *h_d_* = 1.45 mm, e.g., *h_d_* = 1.60 mm, resulted in a reduction in the maximum load capacity of the joint in the shear test by 2.5%, and for *h_d_* = 1.75 mm, it reduced the maximum average shear force of the joint by 3.5%. The forming force for *o_f_* = 0 mm was reduced by 12% by increasing the die depth from 1.45 mm to 1.75 mm (Figure 9 and Figure 10). Instead of increasing the size of the additional rivet, the strength of the clinch joint can be increased using local thermal heating through resistance welding [7,46]. However, this requires a special stand and modified tools. This method of increasing the load capacity of the joints causes thermal changes in the structure of the material.

**Figure 20:**
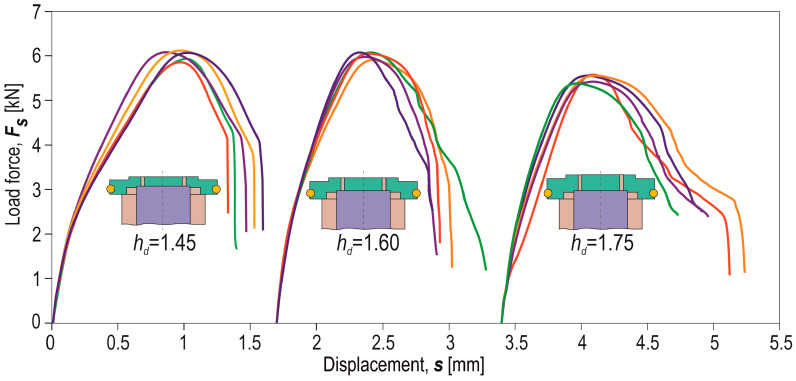
Examples of the forming force–displacement diagrams for “CR” joints formed with different die depths (*h_d_* in mm) and offset value *o_f_* = 0 mm.

**Figure 21:**
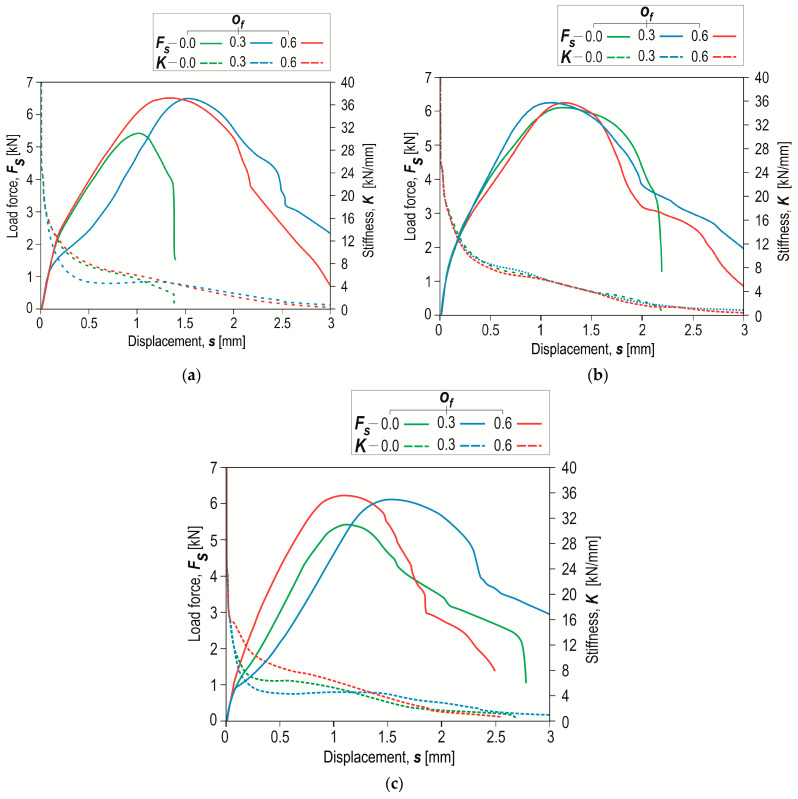
Examples of the forming force–displacement diagrams and stiffness–displacement diagrams for “CR” joints formed with the depth of the die, *h_d_*, equal to (**a**) 1.45 mm, (**b**) 1.60 mm, (**c**) 1.75 mm.

Examples of shear force–displacement diagrams for a series of 5 samples of joints formed with different die depths (*h_d_* = 1.45, 1.60 and 1.75 mm) and offset *o_f_* = 0 mm are presented in Figure 20. The course of the shear force–displacement diagrams was very similar, but the maximum joint shear force was different. In the initial phase of loading the joint, the curves had slightly different angles in the linear section. Therefore, it was decided to compare the curves of the average shear force–displacement diagrams and the stiffness–displacement diagrams of the “CR” joint (Figure 21).

The use of an additional rivet can be used to strengthen or repair a damaged clinch joint [24]. The authors showed that increasing the pressing force of a special tubular rivet resulted in an increase in the size of the interlock and the load capacity of the joint. In addition to the maximum load capacity of the pressed joints, the aspect of joint stiffness is extremely important. In one of the works including research on joint stiffness [47], the authors analysed the load parameters and joint stiffness for a combination of aluminium-alloy and copper-alloy materials. These are low-strength materials. HX340 steel sheets are a group of materials with increased strength that are increasingly used in car bodies [48].

**Figure 22:**
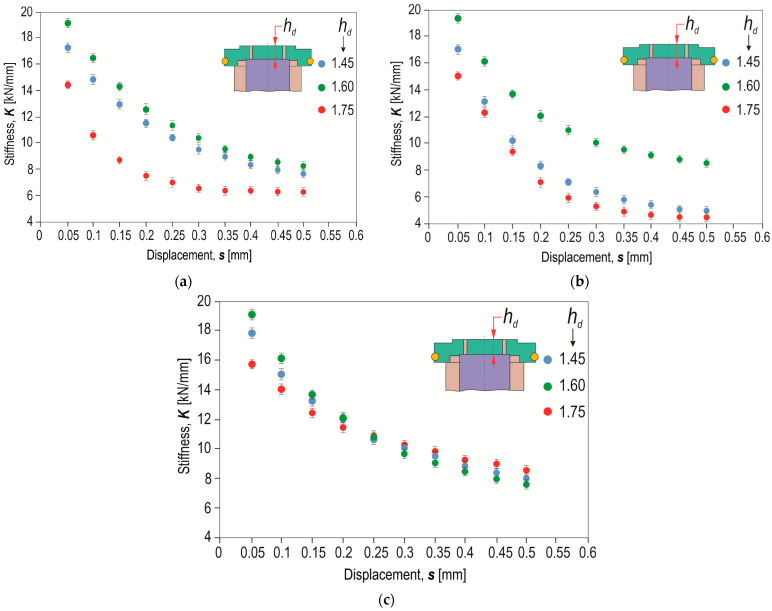
The influence of the depth of the die (*h_d_*) on the joint stiffness for different values of displacement for the following offsets *o_f_*: (**a**) 0 mm, (**b**) 0.3 mm, (**c**) 0.6 mm.

To analyse the influence of the depth of the die (for a constant offset value) on the change in joint stiffness, the calculated joint stiffness values for three values of *h_d_* (=1.45, 1.60 and 1.75 mm) are presented (Figure 22a). The joint made using a die with *h_d_* = 1.60 mm was characterized by the highest stiffness. Increasing the depth of the die from 1.45 mm to 1.6 mm resulted in an increase in stiffness. For joints made using a die of *h_d_* = 1.75 mm, a decrease in joint stiffness was observed. In each of the analysed cases, the highest joint stiffness was obtained from the offset values *o_f_* = 0, 0.3 and 0.6 mm to a displacement of 0.2 mm (Figure 22). When the offset value *o_f_* = 0.3 mm was used, the difference in stiffness increased as the displacement increased (Figure 22b). For the highest offset value, *o_f_* = 0.6 mm, the differences between stiffnesses for joints formed using a die of different depth were the smallest (Figure 22c).

**Figure 23:**
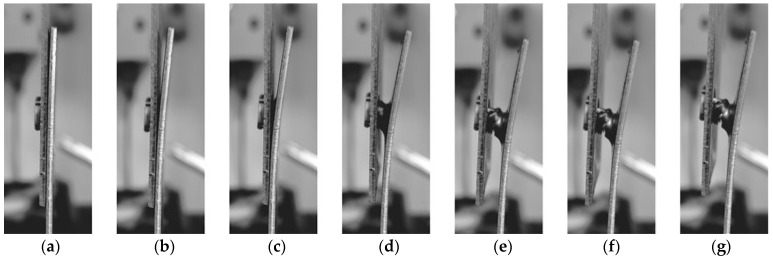
Examples of the samples ‘deformation stages during tensile shear test (**a**–**g**) (*o_f_* = 0.6 mm, *h_d_* = 1.45 mm).

**Figure 24:**
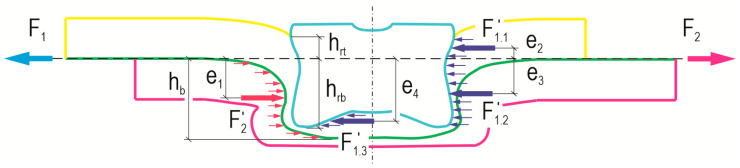
Scheme of the forces acting during the tensile shear test of the “CR” joint.

When a joint is carrying a load, there is a specific system of forces acting on the internal surfaces of the joint. These forces create a torque moment that causes the rivet to rotate in the joint. As the load increases, the sheets bend, and the rivet is pulled out of the lower sheet (Figure 23 and Figure 24). Figure 23a–g shows an example of the behaviour of the joint elements during a shear test of a lap joint of 1.5 mm thick sheets made of HX340 steel. 

When joining, for example, aluminium alloys, smaller deformations of the sheets are observed during the shear test [49]. The authors presented the possibility of the two-stage formation of a clinch joint with an additional deformable rivet. Unfortunately, such a process is more complex, time-consuming and expensive than the conventional clinch-rivet joining process.

Although the plane of action of the force loading the lap joint was in the common plane of the sheets during the shear test, the sheets were bent. During a shear test of a lap joint, there is a certain system of forces relative to the plane of load action (Figure 24). Part of the height of the rivet (*h_b_*) relative to the common surface of the sheets is greater in the lower sheet (Figure 24). The rivet is more blocked on the lower sheet and creates resistance so that it causes greater bending of the upper sheet (in Figure 23—right sheet). Forces *F*_1_ and *F*_2_ act on the joint in one plane. The embossment surfaces of the sheets that carry the load are not uniformly located in relation to the line of load action. The force *F*_2_*’* acts on the distance *h_b_* relative to the line of action of the load forces:

(1) $$M_{2}=F_{2}^{\prime }\cdot e_{1}.$$

The force *F*_1_ is distributed on the surfaces that are below and above the line of action of the load forces:

$${-M}_{1.3}=F_{1.3}^{\prime }\cdot e_{4},$$

(2) $${-M}_{1.2}=F_{1.2}^{\prime }\cdot e_{3},$$

$$M_{1.1}=F_{1.1}^{\prime }\cdot e_{2}.$$

The forces acting in the tensile shear test are as follows:

(3) $$F_{1}=F_{1.1}^{\prime }+F_{1.2}^{\prime }+F_{1.3}^{\prime },$$

(4) $$F_{2}=F_{2}^{\prime }.$$

**Figure 25:**
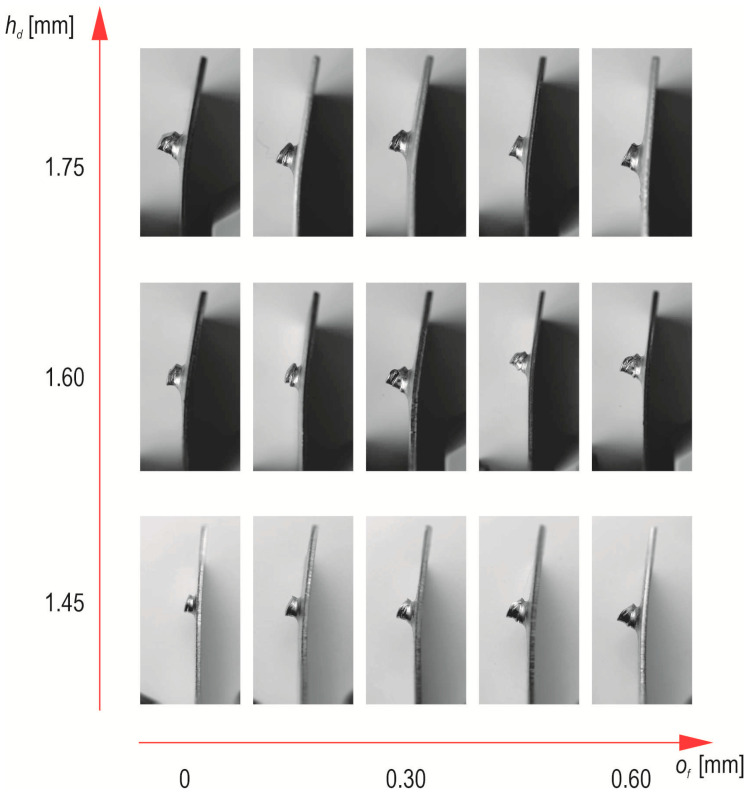
The influence of the depth of the die (*h_d_*) and the offset value (*o_f_*) on the deviations of the sheets after tensile shear test.

The greater the depth of the die (*h_d_*), the greater the reaction moment of internal forces in the joint and the bending of the sheet on the side of the rivet press-in (Figure 25). In the case of using a die with a depth of *h_d_* = 1.45 mm, the greater the offset used, the greater the observed bending of the sheet metal was. The rivet was pressed deeper and blocked more tightly. Increasing the depth of the die caused the rivet to be pressed deeper into the materials being joined. The greater the depth of the die, the greater the bending of the sheet metal (for the case of offset value *o_f_* = 0 mm). The higher the offset value, the smaller the observed degree of bending was for joints formed with a depth greater than *h_d_* = 1.45, i.e., 1.60 or 1.75 mm.

**Figure 26:**
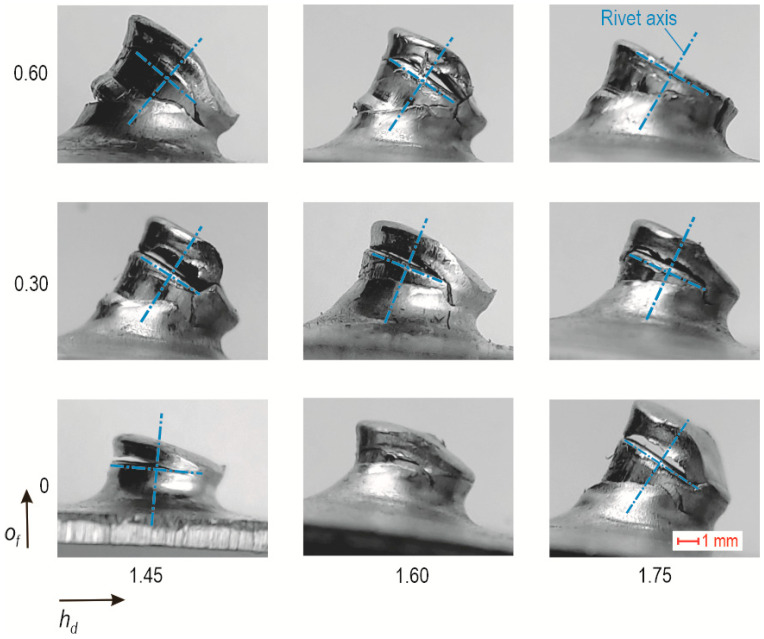
The macrostructure of the “CR” joint after tensile shear with a marked deviation of the joint axis.

To discuss the differences in the behaviour of the rivet in the tensile shear test, the results of some cases of joint formation are presented. For all joint combinations tested, two failure mechanisms occurred during the tensile shear test of the “CR” lap joint (Figure 26). The first and most common was the splitting of the embossment of the upper and lower sheets. The rivet remained in the upper plate, with the embossment partially failing. The embossment of the top sheet failed. The second failure mechanism occurred for the highest offset value and the greatest depth of the die. Part of the embossment of the upper sheet failed and remained in the lower sheet. The greater the offset used, the more the rivet axis was deflected in the lap-joint shear test. In the case of joints formed using a die with *h_d_* = 1.45 mm, it was observed that the higher the offset value of the joint, the greater the deflected rivet axis during the tensile shear test of the lap joint. However, for joints formed with a constant value *o_f_* and a variable value of *h_d_*, it was observed during the tensile shear test that the deflection of the rivet axis decreased when the depth of the die increased.

For the case of the formed joint with *h_d_* = 1.6 mm and *o_f_* = 0 mm in the tensile shear test, it was not possible to determine the deviation of the rivet axis (the rivet was not exposed from the upper sheet). 

## 4. Conclusions

The presented research shows the influence of the use of different rivet insertion depths on the strength of the joint formed using a die of different depths *h_d_* = 1.45, 1.60 and 1.75 mm. It is possible to strengthen a “CR” joint by changing the pressing depth of the steel rivet without disturbing the cohesion of the sheet material. However, the use of a higher rivet press value resulted in a significant increase in forming force and energy consumption. The significant and most important information obtained from the conducted research is as follows:

1. As the depth of the die (*h_d_*) increased, the maximum value of the forming force decreased. Increasing the depth of the die from 1.45 mm to 1.75 mm (by more than 20%) resulted in a reduction in the forming force by 12%. The greater the rivet offset below the upper surface of the sheet metal, the smaller the difference in the forming force.
2. The lesser the die depth (*h_d_*) used, the greater the maximum shear force obtained in the lap joint. The size of the rivet offset below the surface of the upper sheet increased by 13%, while the maximum joint load capacity increased by only 7% (*h_d_* = 1.45 mm). In the case of increasing the die depth to 1.75 mm, an increase of 0.6 mm in the offset value resulted in an increase in the forming force by 10%, and the maximum joint shear force increased by 6.5%.
3. Increasing the value of the rivet offset resulted in an increase in the load capacity of the joint. Analysing the geometry of the interlock, it was observed that increasing the offset from 0 mm to 0.6 mm resulted in an increase in the interlock *t_u_* (mm). For a joint formed using a die with *h_d_* = 1.45 mm, the interlock value (*t_u_*) increased by 136%; for *h_d_* = 1.60 mm, by almost 128%; and for *h_d_* = 1.75 mm, the interlock value increased by 85%.
4. Despite a relatively large increase in the size of the interlock (*t_u_*), there was no significant increase in the maximum shear force. A change in the stiffness of the connection was observed. Increasing the depth of the die from 1.45 mm to 1.60 mm (an increase of over 10%) resulted in an increase of 12%in the joint stiffness value. In turn, increasing the depth of the die by more than 20% (to 1.75 mm) resulted in the joint having a 16% lower stiffness in the initial loading phase (for a joint formed with a die with *h_d_* = 1.45 mm).
5. Analysis of the deformation of the sheet-metal surface in the area of the joint showed that for each value of the position of the upper plane of the rivet in the joint, it was below the given value (measured in relation to the nominal model). The largest deformations were obtained in the area analysed for the joints formed with a *h_d_* = 1.45 mm. As a result of the impact of springback deformations, the upper surface of the rivet in the joint moved by almost 0.23 mm (*o_f_* = 0 mm, whereas for *o_f_* = 0.6, it was 102% lower than the set position. With an increase in the offset *o_f_*, the deformation of the sheets and the area of the deformation changed. Increasing the rivet offset depth from 0 mm to 0.6 mm resulted in a deviation of the sheets in the analysed area by a maximum of 0.3 mm.

## Figures and Tables

**Table 1:** Chemical composition of HX340LAD+Z sheets (maximum percentage by weight [%]).

Mn	Si	Ti	C	Nb	P	S	Al	Fe
0.88	0.01	0.011	0.09	0.031	0.02	0.007	0.032	remainder

**Table 2:** Mechanical properties of HX340LAD+Z sheets.

Material Designation	Surface Finish + Z [g/m^2^]	Young’s Modulus E [GPa]	Poisson’s Ratio ν	Yield Strength R_p0.2_ [MPa]	Tensile Strength R_m_ [MPa]	Elongation after Fracture A_80_ [%]	Strength Coefficient K [MPa]	Strain Hardening Exponent n [–]
HX340	zinc layer quality 100	188	0.29	365	463	29	807	0.16

**Table 3:** The list of “CR” joint variants.

Offset Dimension *o_f_* mm	Die Depth *h_d_* mm		
	1.45	1.60	1.75
0	I-00	II-00	III-00
0.15	I-15	II-15	III-15
0.30	I-30	II-30	III-30
0.45	I-45	II-45	III-45
0.60	I-60	II-60	III-60

## Data Availability

Data available on request due to restrictions.
